# Treatment Outcomes of Clofazimine-Containing Regimens in Severe *Mycobacterium avium* Complex Pulmonary Disease

**DOI:** 10.1093/ofid/ofad682

**Published:** 2023-12-28

**Authors:** Inhan Lee, Eui Jin Hwang, Joong-Yub Kim, Jae-Joon Yim, Nakwon Kwak

**Affiliations:** Division of Pulmonary and Critical Care Medicine, Department of Internal Medicine, Seoul National University College of Medicine, Seoul, South Korea; Department of Radiology and Institute of Radiation Medicine, Seoul National University College of Medicine, Seoul, South Korea; Division of Pulmonary and Critical Care Medicine, Department of Internal Medicine, Seoul National University College of Medicine, Seoul, South Korea; Division of Pulmonary and Critical Care Medicine, Department of Internal Medicine, Seoul National University College of Medicine, Seoul, South Korea; Division of Pulmonary and Critical Care Medicine, Department of Internal Medicine, Seoul National University College of Medicine, Seoul, South Korea

**Keywords:** clofazimine, *Mycobacterium avium* complex, outcomes

## Abstract

**Background:**

Clofazimine is suggested as a promising drug for the treatment of nontuberculous mycobacterial pulmonary disease. However, the role of clofazimine in severe *Mycobacterium avium* complex pulmonary disease (MAC-PD) remains unclear. In this study, we investigated the treatment outcomes of patients with severe MAC-PD treated with regimens containing clofazimine.

**Methods:**

This study included patients diagnosed with severe MAC-PD at Seoul National University Hospital who underwent anti-mycobacterial treatment between 1 January 2011 and 31 December 2022. We assessed the rate of culture conversion within 6 months and microbiological cure in patients receiving clofazimine-containing regimens, considering the dose and duration of clofazimine administration.

**Results:**

A total of 170 patients with severe MAC-PD, treated with regimens containing clofazimine, were included in the analysis. The median age of patients was 68 years (interquartile range, 59–75 years), with a female predominance (n = 114 [67.1%]). Cavities were identified in 121 patients (71.2%). Within 6 months, 77 patients (45.3%) achieved culture conversion, and 84 of 154 (54.6%) patients attained microbiological cure. The dose of clofazimine (100 mg vs 50 mg) was not associated with culture conversion (adjusted odds ratio [aOR], 0.64 [95% confidence interval {CI}, .29–1.42]) or microbiological cure (aOR, 1.21 [95% CI, .52–2.81]). The microbiological cure rate reached 71.0% when clofazimine was administered for 6–12 months, compared to 23.1% when administered for <6 months.

**Conclusions:**

Clofazimine demonstrated a relatively favorable efficacy in severe MAC-PD, regardless of the maintenance dose. This effect was more pronounced when administered for a duration exceeding 6 months.

## Teaser

Clofazimine demonstrated a favorable treatment outcome in severe *Mycobacterium avium* complex pulmonary disease. There was no significant difference in outcomes between maintenance doses of 50 mg and 100 mg. Patients who received clofazimine for >6 months presented higher microbiological cure rates.

Nontuberculous mycobacteria (NTM), which encompass mycobacteria other than *Mycobacterium tuberculosis* or *Mycobacterium leprae*, cause chronic infections in humans, with pulmonary disease (PD) being the most common manifestation [1]. While the burden of NTM-PD has increased [2], the treatment of NTM-PD is still challenging. The treatment of *Mycobacterium avium* complex pulmonary disease (MAC-PD), which constitutes about 75% of NTM-PD [3], generally requires the administration of several antibiotics for at least 12 months following the achievement of culture conversion [1]. However, the treatment success rate remains approximately 60% [4].

In cavitary or moderate-to-severe MAC-PD, the current guidelines recommend the administration of amikacin at an early phase [1, 5]. Amikacin exhibits notable in vitro activity against clinical isolates of MAC through its binding to 16S ribosomal RNA (rRNA) within the bacterial 30S ribosomal subunit [6]. Subsequently, amikacin leads to clinical responses in about 75% of patients with NTM-PD [7]. However, the long-term administration of amikacin unavoidably leads to serious adverse events. Nephrotoxicity and ototoxicity occur in 6.3% and 25.0% of patients receiving amikacin, respectively [8].

Clofazimine, a phenazine molecule, inhibits mycobacteria's respiratory chains and ion transporters [9]. When combined with clarithromycin or amikacin, clofazimine exhibits a synergistic effect against *M avium* and *Mycobacterium abscessus* [10]. Due to these potent anti-mycobacterial effects, clofazimine has emerged as a repurposing drug for NTM-PD. In MAC-PD, clofazimine-containing regimens have demonstrated a significantly higher culture conversion rate than rifampicin-containing regimens [11]. Nevertheless, the ideal dose of clofazimine remains elusive [1, 5]. Furthermore, given the frequent occurrence of adverse events [12], the optimal duration for clofazimine administration remains unknown.

In this study, we analyzed the treatment outcomes of patients treated with clofazimine-containing regimens in severe MAC-PD. Through this analysis, we aim to provide insights into the appropriate usage of clofazimine.

## METHODS

### Study Design and Participants

This retrospective study included patients who were diagnosed as MAC-PD at Seoul National University Hospital, following the criteria suggested by the American Thoracic Society/European Respiratory Society/European Society of Clinical Microbiology and Infectious Diseases Society of America [1] and the British Thoracic Society [5]. We included patients who underwent anti-mycobacterial treatment between 1 January 2011 and 31 December 2022. This study was conducted in accordance with the amended Declaration of Helsinki. The Institutional Review Board (IRB) of Seoul National University Hospital approved the protocol (IRB number 2303-167-1416).

### Treatment Protocol

The composition of the treatment regimen was primarily determined based on clinical guidelines and the severity of MAC-PD [1, 5]. The regimens in our institution should include macrolide (preferably azithromycin 250 mg daily or clarithromycin 500 mg twice daily) and ethambutol (15 mg/kg daily) unless contraindicated. The decision to use rifampicin (10 mg/kg daily) is made based on physicians’ judgment, taking into consideration potential adverse events and drug interactions [13]. If acid-fast bacilli (AFB) smears were positive, cavities were present, or severe symptoms/systemic illness were observed [5], intravenous amikacin was primarily recommended. Amikacin was also recommended once the attending physicians judged the extent of the disease to be moderate or greater, based on the evaluation of chest computed tomography (CT) scans. Typically, if the severity of bronchiectasis (bronchus diameter ≥2 times the vessel diameter) or cellular bronchiolitis (involving >1–3 cm from the pleura or extending into the central lung) was moderate or severe; if the extent of bronchiectasis, mucus plugging, cellular bronchiolitis, or nodules was ≥6 segments; or if the consolidation involved ≥3 segments [14], these patients were advised to receive amikacin. However, depending on the patient's adherence and preference and physicians’ judgment, inhaled amikacin or clofazimine was also considered. In our institution, clofazimine was generally administered at 50 mg per day for individuals weighing ≤50 kg and 100 mg per day for those weighing >50 kg. The duration of clofazimine administration was determined according to treatment responses and adverse events. In general, treatment was sustained for a minimum of 12 months after achieving culture conversion. However, at the discretion of attending physicians, the treatment could be extended.

This study analyzed patients who were determined as severe MAC-PD (AFB smear positive, radiographic evidence of cavity, severe symptoms/systemic illness, or moderate or greater disease extent judged by the attending physicians based on chest CT findings) and received clofazimine instead of amikacin as part of their initial regimen within the first 3 months of treatment initiation.

### Clinical Data

The demographic and clinical characteristics were collected. The variables included age, sex, body mass index (BMI), history of prior NTM-PD or tuberculosis, and comorbidities such as diabetes, chronic kidney disease, chronic liver disease, and chronic obstructive pulmonary disease. Radiographic findings (radiographic types, presence of cavities, and their size) were assessed by 3 reviewers (2 pulmonologists [I. L. and N. K.] and 1 chest radiologist [E. J. H.]). The size of the cavity was determined by measuring the longest diameter on axial images. Three measurements were taken, and the average value was recorded [15]. Data on AFB smear, mycobacterial cultures, mycobacterial species identified using 16S rRNA and *rpo*B gene sequencing [16–18], and drug susceptibility test results were also obtained.

### Outcomes

Treatment outcomes were assessed based on the definitions provided by the NTM-NET consensus statement [19]. Culture conversion was defined as at least 3 consecutive negative mycobacterial cultures from respiratory samples collected at least 4 weeks apart. Microbiological cure was determined when culture conversion was achieved and maintained until the end of anti-mycobacterial treatment. Sputum samples were usually collected at 1-month intervals following the initiation of treatment. Adverse drug reactions (ADRs) necessitating the discontinuation of clofazimine were documented, encompassing skin pigmentation, gastrointestinal discomfort, hepatotoxicity, and QTc prolongation. Hepatotoxicity was defined as follows: (*i*) elevation of either alanine transaminase, aspartate aminotransferase, and/or bilirubin >5 times the upper limit of normal (ULN), or (*ii*) 3 times the ULN with concurrent clinical symptoms [13]. Discontinuation of clofazimine occurred if the QTc interval was >500 milliseconds at any point in time or increased by >60 milliseconds from baseline [20]. The attribution of causality between clofazimine and ADRs was determined through a consensus between the attending physicians and the investigators.

### Statistical Analysis

Data are represented as median values with interquartile ranges (IQRs) for continuous variables and as proportions for categorical variables. The Wilcoxon rank-sum and Fisher exact tests were used to compare continuous and categorical variables, respectively. Univariate logistic regression analysis was conducted to determine the factors associated with culture conversion and microbiological cure. For multivariate analysis, variables with a statistical significance of *P* < .1 on univariate analysis were included. *P* values < .05 were considered statistically significant. All analyses were conducted using Stata version 17.0 (StataCorp, College Station, Texas).

## RESULTS

### Baseline Characteristics

During the study period, a total of 290 patients with severe MAC-PD were treated with clofazimine (n = 170), amikacin (intravenous or inhaled) (n = 40), or both (n = 80). Because 2 medications delivered concurrently or sequentially can confound the effect of the target drug, we only included 170 patients who received clofazimine alone for this analysis. The median age of patients was 68 years (IQR, 59–75 years), and most were female (n = 114 [67.1%]). Fifty-eight patients (34.1%) had a history of prior treatment of NTM-PD, and there were no people with human immunodeficiency virus. Nine patients (5.3%) were infected with macrolide-resistant strains. Detailed drug susceptibility results are presented in Supplementary Table 1. The most prevalent radiographic form was the cavitary nodular bronchiectatic type (n = 75 [44.1%]), followed by the noncavitary nodular bronchiectatic type (n = 49 [28.8%]) and the fibrocavitary type (n = 46 [27.1%]). While cavities were confirmed in 121 patients (71.2%), 76 patients had a cavity diameter of 2 cm or more. *Mycobacterium intracellulare* (n = 84 [49.4%]) was the most common causative species, followed by *M avium* (n = 62 [36.5%]).

Macrolide was administered to 169 patients (168 with azithromycin, 1 with clarithromycin). Ethambutol was prescribed to 150 patients (88.2%), excluding 20 individuals who had previously experienced ADRs related to ethambutol. Rifampicin was administered to 15 patients (8.8%). Sixty-eight patients (40.0%) received 50 mg per day of clofazimine, while 102 patients (60.0%) received 100 mg daily. Patients receiving 50 mg per day had a median weight of 44.9 kg (IQR, 39.9–50.3 kg) and a BMI of 18.0 kg/m^2^ (IQR 16.6–21.3 kg/m^2^), which were lower than the weight (54.9 kg [IQR, 49.1–59.0 kg], *P* < .001) and BMI (21.0 kg/m^2^ [IQR, 19.2–22.7 kg/m^2^], *P* = .001) of patients receiving 100 mg per day. Detailed baseline characteristics are presented in Table 1.

**Table 1.** Baseline Characteristics of Patients According to Treatment Regimen

Characteristic	Total (N = 170)	Maintenance Dose		
		50 mg (n = 68)	100 mg (n = 102)	*P* Value^a^
Age, y, median (IQR)	68 (59–75)	72 (65–78)	64 (58–73)	<.001
Male sex	56 (32.9)	16 (23.5)	40 (39.2)	.033
Body weight, kg, median (IQR)	50.8 (44.0–56.6)	44.9 (39.9–50.3)	54.9 (49.1–59.0)	<.001
BMI, kg/m^2^, median (IQR)	20.0 (17.7–22.4)	18.0 (16.6–21.3)	21.0 (19.2–22.7)	.001
History of tuberculosis	30 (17.6)	17 (25.0)	13 (12.7)	.040
Comorbidities				
Diabetes mellitus	23 (13.5)	11 (16.2)	12 (11.8)	.410
Chronic kidney disease	12 (7.1)	6 (8.8)	6 (5.9)	.546
COPD	7 (4.1)	2 (2.9)	5 (4.9)	.704
Chronic liver disease	8 (4.7)	2 (2.9)	6 (5.9)	.478
Presence of cavity ≥2 cm	76 (44.7)	26 (38.2)	50 (49.0)	.166
Smear positivity	38 (22.4)	15 (22.1)	23 (22.5)	.940
*Mycobacterium* spp				.507
*M avium*	62 (36.5)	28 (41.2)	34 (33.3)	
*M intracellulare*	84 (49.4)	30 (44.1)	54 (52.9)	
Others	24 (14.1)	10 (14.7)	14 (13.7)	

Data are presented as No. (%) unless otherwise indicated.

Abbreviations: BMI, body mass index; COPD, chronic obstructive pulmonary disease; IQR, interquartile range.

^a^The *P* value was calculated based on the comparison of maintenance doses of clofazimine.

### Treatment Outcomes and ADRs

The median duration of treatment was 18.6 months (IQR, 12.4–25.8 months), with clofazimine being administered for a median of 13.3 months (IQR, 8.6–20.6 months). Seventy-seven patients (45.3%) achieved culture conversion within 6 months after the initiation of treatment. Microbiological cure was achieved in 84 of 154 (54.6%) patients whose treatment outcomes could be evaluated. Among 84 patients, 7 (8.3%) experienced recurrence or relapse within a year after treatment completion. The proportion of culture conversion (*P* = .348) and microbiological cure (*P* > .999) was not affected by the maintenance dose of clofazimine (Table 2).

**Table 2.** Treatment Outcomes and Adverse Events

Outcome	Total (N = 170)	Maintenance Dose		
		50 mg (n = 68)	100 mg (n = 102)	*P* Value^a^
Total treatment duration, median (IQR)	18.6 (12.4–25.8)	17.1 (10.9–22.4)	19.7 (12.6–29.4)	.015
Clofazimine use duration, median (IQR)	13.3 (8.6–20.6)	12.8 (8.7–18.2)	13.8 (8.5–22.1)	.130
Culture conversion within 6 mo	77 (45.3)	34 (50.0)	43 (42.2)	.348
Microbiological cure^b^	84/154 (54.6)	33/61 (54.1)	51/93 (54.8)	>.999
Adverse drug reactions necessitating the discontinuation of clofazimine	25 (14.7)	9 (13.2)	16 (15.7)	.658
Skin pigmentation	9 (5.3)	3 (4.4)	6 (5.9)	.743
Hepatotoxicity	8 (4.7)	3 (4.4)	5 (4.9)	>.999
Gastrointestinal discomfort	6 (3.5)	3 (4.4)	3 (2.9)	.684
QTc prolongation	2 (1.2)	0 (0.0)	2 (2.0)	.517

Data are presented as No. (%) unless otherwise indicated.

^a^The *P* value was calculated based on the comparison of maintenance doses of clofazimine.

^b^Microbiological cure was assessed in 154 of 170 patients.

During the treatment period, 25 patients (14.7%) discontinued the use of clofazimine due to skin pigmentation (n = 9 [5.3%]), hepatotoxicity (n = 8 [4.7%]), gastrointestinal discomfort (n = 6 [3.5%]), and QTc prolongation (n = 2 [1.1%]). There was no difference in the frequency of ADRs based on the dosage of clofazimine (*P* = .658).

### Impact of Clofazimine Dosage on Treatment Outcomes

In the univariate analysis, older age, male sex, lower BMI, smear positivity, presence of cavity >2 cm, and the isolation of *M intracellulare* predicted a failure in culture conversion within 6 months. Likewise, older age, male sex, and smear positivity were associated with a failure in microbiological cure (Supplementary Table 2). However, in the multivariate analysis, only smear positivity was inversely associated with microbiological cure (adjusted odds ratio [OR], 0.34 [95% confidence interval {CI}, .13–.86]). The dose of clofazimine (100 mg vs 50 mg) did not affect the culture conversion within 6 months (adjusted OR, 0.64 [95% CI, .29–1.42]) or microbiological cure (adjusted OR, 1.21 [95% CI, .52–2.81]) (Table 3).

**Table 3.** Predictive Factors for Culture Conversion Within 6 Months and Microbiological Cure (Multivariate)

Factor	Culture Conversion Within 6 Months, aOR (95% CI)	*P* Value	Microbiological Cure, aOR (95% CI)	*P* Value
Age, y	0.97 (.94–1.01)	.118	0.98 (.94–1.01)	.196
Male sex	0.83 (.38–1.82)	.636	0.44 (.19–1.05)	.064
BMI, kg/m^2^	1.10 (.98–1.23)	.116	0.99 (.89–1.11)	.888
COPD	0.33 (.04–3.21)	.342	0.23 (.20–2.64)	.239
Smear positivity	0.65 (.27–1.59)	.347	0.34 (.13–0.86)	.023
Presence of cavity >2 cm	0.85 (.40–1.79)	.665	0.89 (.40–1.99)	.776
*Mycobacterium* spp				
*M avium*	Reference		Reference	
*M intracellulare*	0.58 (.26–1.28)	.177	1.16 (.49–2.75)	.735
Others	0.82 (.30–2.25)	.692	1.62 (.51–5.19)	.416
Clofazimine maintenance dose				
50 mg	Reference		Reference	
100 mg	0.64 (.29–1.42)	.275	1.21 (.52–2.81)	.667

Abbreviations: aOR, adjusted odds ratio; BMI, body mass index; CI, confidence interval; COPD, chronic obstructive pulmonary disease.

### Treatment Outcomes Based on the Maintenance Duration of Clofazimine

Clofazimine was used for <6 months in 27 patients owing to ADRs by clofazimine (11 patients), patient preference (5 patients), death (3 patients), high minimum inhibitory concentration (MIC) of clofazimine (1 patient), and other reasons (7 patients). When clofazimine was administered for <6 months, the rate of culture conversion within 6 months was 14.8%, significantly lower than in patients who received clofazimine for 6 months or longer (*P* < .001). Similarly, the microbiological cure was the lowest (23.1%) in patients who received clofazimine for <6 months. However, in cases where clofazimine was administered for 6–12 months, the rate of culture conversion within 6 months was 42.5%, with a microbiological cure rate of 71.0%. Details of treatment outcomes based on the duration of clofazimine use are provided in Table 4.

**Table 4.** Treatment Outcomes According to the Duration of Clofazimine Use

Outcome	<6 Months (n = 27)	6–12 Months (n = 40)	12–18 Months (n = 41)	>18 Months (n = 62)	*P* Value
Culture conversion, No. (%)					
50 mg per day	2 (20.0)	9 (45.0)	14 (70.0)	9 (50.0)	.081
100 mg per day	2 (11.8)	8 (40.0)	14 (66.7)	19 (43.2)	.007
Total	4 (14.8)	17 (42.5)	28 (68.3)	28 (45.2)	<.001
Microbiological cure^a^, No. (%)					
50 mg per day	1/9 (11.1)	11/15 (73.3)	11/19 (57.9)	10/18 (55.6)	.030
100 mg per day	5/17 (29.4)	11/16 (68.8)	13/19 (68.4)	22/41 (53.7)	.074
Total	6/26 (23.1)	22/31 (71.0)	24/38 (63.2)	32/59 (54.2)	.002

^a^Microbiological cure was assessed in 154 of 170 patients.

## DISCUSSION

This study aimed to assess the therapeutic effects of combining clofazimine with macrolide/ethambutol in patients with severe MAC-PD. In our study, clofazimine exhibited lower initial bacterial conversion and microbiological cure rates. Notably, the outcomes did not show significant differences based on the dose of clofazimine. However, when the use of clofazimine was extended beyond 6 months, the treatment outcomes improved. These results suggest that clofazimine could play a central role in treating severe MAC-PD, particularly when used for >6 months.

The efficacy of amikacin in treating NTM-PD has been established through various in vitro and in vivo studies [21, 22]. Clinical guidelines have recommended amikacin usage for patients with conditions such as cavities, severe extent, or treatment refractoriness [1, 5]. However, the use of amikacin often leads to irreversible hearing or renal impairment. Recently, the amikacin liposome inhalation suspension (ALIS), designed to enhance absorption into alveolar macrophages, has been developed. However, ALIS was also associated with treatment-emergent adverse events, including dysphonia, cough, and hemoptysis in 17.5% of patients [23]. Consequently, there is an urgent need for all-oral regimens to treat severe MAC-PD, ensuring they are both effective and well-tolerated.

In this study, the regimens comprising macrolide, ethambutol, and clofazimine exhibited a treatment success rate of approximately 55% in patients with severe MAC-PD. The results were comparable to those of conventional regimens [4, 24]. However, unfavorable prognostic factors, such as older age, large cavities, and *M intracellulare* infection, were prevalent in these patients [25–27]. Given the treatment success rate of about 60% in patients with cavitary MAC-PD treated with aminoglycoside [28], clofazimine could be a viable alternative treatment option for severe MAC-PD when amikacin is not feasible.

One of the crucial issues using clofazimine in mycobacterial infections is determining the appropriate dosage. The recommended dose ranges from 50 to 200 mg daily [1, 5, 29]. However, in our study, 40.0% of patients received a dosage of 50 mg. Notably, treatment outcomes did not differ between patients receiving 50 mg and those receiving 100 mg. According to a study from South Africa, when clofazimine was administered at a dosage of 100 mg, the estimated steady-state concentration was reported to be 0.36 mg/L, greater than the putative target concentration of 0.25 mg/L [30]. However, a study from Japan revealed that administering 50 mg of clofazimine resulted in a target concentration of 0.86 mg/L in NTM-PD [31]. A plausible explanation for the favorable outcomes with low-dose clofazimine might be that patients receiving 50 mg had a lower body weight than those receiving 100 mg. Higher body weight increased the clearance of clofazimine [31]. Therefore, administering 50 mg of clofazimine might be reasonable for patients weighing ≤50 kg.

In this study, the rates of culture conversion and microbiological cure were higher when clofazimine was used for >6 months than when it was used for a shorter period. Clofazimine requires approximately 5 months to reach a steady state owing to its extended half-life [31]. As a result, the microbiologic cure rate was only 23.1% in patients treated with clofazimine for <6 months, while patients treated for >6 months showed a cure rate ranging from 54.2% to 71.0%, which can be explained by the longer sustainment of clofazimine. On the other hand, in patients who received clofazimine for >18 months, prolonged use of clofazimine might have been necessary due to unfavorable treatment responses.

The incidence of ADRs leading to clofazimine discontinuation varies across studies, ranging from 6.5% to 42.9% [11, 32]. In our study, which included the largest number of patients treated with clofazimine compared with other studies, 25 (14.7%) of 170 patients discontinued clofazimine due to ADRs, confirming its relatively favorable safety profile. In particular, hepatotoxicity was comparatively high, while gastrointestinal discomfort was less frequent than in previous studies [32, 33]. Further research is imperative to fully understand the ADRs associated with clofazimine.

Although clinical guidelines recommend rifampicin for MAC-PD, the use of rifampicin is associated with frequent adverse events [34] and a decrease in macrolide concentrations [35]. Furthermore, treatment outcomes with a regimen without rifampicin were comparable to those with rifampicin [36]. Based on these findings, our institution favors regimens without rifampicin [13]. Of note, rifampicin was administered to only 15 of the 170 patients in this study. Given the relatively favorable outcomes observed in our study, the regimens consisting of macrolide, ethambutol, and clofazimine—predominantly used in combination in this study—can be a reasonable option for severe MAC-PD.

This study has several limitations. First, the analysis excluded 80 patients who received both clofazimine and amikacin, leading to a risk of selection bias in our study population. Combining clofazimine and amikacin could potentially lead to a synergistic effect [10]. However, we aimed to directly demonstrate the potential of clofazimine as a substitute for amikacin in the treatment of severe MAC-PD. Groups receiving both agents might have had the most extensive disease, which makes the analysis more complex. Second, it was not possible to adjust the MIC of clofazimine in this study. The measurement of MIC for clofazimine, which has been associated with treatment outcomes [27], has only been implemented in clinical settings since 2022. As a result, pretreatment MIC values for clofazimine were available for only 49 patients. The investigation into the impact of MIC values on treatment outcomes and any alterations observed during clofazimine administration will be conducted in a subsequent study.

In conclusion, clofazimine demonstrated a relatively favorable efficacy in severe MAC-PD. Notably, this performance was more pronounced when clofazimine was used for >6 months. Therefore, clofazimine could be a promising therapeutic option in severe MAC-PD.

## Supplementary Material

ofad682_Supplementary_Data

## Supplementary Data

Supplementary materials are available at *Open Forum Infectious Diseases* online. Consisting of data provided by the authors to benefit the reader, the posted materials are not copyedited and are the sole responsibility of the authors, so questions or comments should be addressed to the corresponding author.

## Notes

***Author contributions*.** Conceptualization: N. K. Data collection: N. K. and I. L. Data analysis and interpretation: N. K., I. L., E. J. H., J.-Y. K., and J.-J. Y. Drafting and editing: N. K., I. L., and J.-Y. K. Supervision: N. K. and J.-J. Y. All the authors have read and agreed to the final version of this manuscript.

***Data availability*.** Access to the data in this study will be considered by the corresponding author upon reasonable request.

***Patient consent*.** The study protocol was approved, and the need to obtain informed consent was waived by the institutional review board at Seoul National University Hospital (approval number 2303-167-1416).
