# Supplementary material for: Treatment Outcomes of Clofazimine-Containing Regimens in Severe Mycobacterium avium Complex Pulmonary Disease
Source: Open Forum Infect Dis. 2023 Dec 28;11(2):ofad682. doi: 10.1093/ofid/ofad682 (PMC10849115; doi:10.1093/ofid/ofad682)
Supplement: ofad682_Supplementary_Data [file ofad682_supplementary_data.docx]

Supplementary Table 1. Baseline minimal inhibitory concentrations of clarithromycin, ethambutol, rifampicin, amikacin, and clofazimine for clinical isolates

|  | Total (N=156) | Maintenance dose | | |
| --- | --- | --- | --- | --- |
|  |  | 50 mg (N=64) | 100 mg (N=92) | *P*-value^*^ |
| Clarithromycin, N (%) |  |  |  | 0.259 |
| ≤ 8 μg/ml | 145 (93.0) | 59 (92.2) | 86 (93.5) |  |
| 16 μg/ml | 2 (1.2) | 2 (3.1) | 0 |  |
| ≥ 32 μg/ml | 9 (5.8) | 3 (4.7) | 6 (6.5) |  |
| Ethambutol, N (%) |  |  |  | 0.647 |
| ≤ 4 μg/ml | 23 (14.7) | 8 (12.5) | 15 (16.3) |  |
| ≥ 8 μg/ml | 133 (85.3) | 56 (87.5) | 77 (83.7) |  |
| Rifampicin, N (%) |  |  |  | 0.745 |
| ≤ 4 μg/ml | 88 (56.4) | 35 (54.7) | 53 (57.6) |  |
| ≥ 8 μg/ml | 68 (43.6) | 29 (45.3) | 39 (42.4) |  |
| Amikacin, N (%) |  |  |  | 0.602 |
| ≤ 16 μg/ml | 124 (79.5) | 53 (82.8) | 71 (77.2) |  |
| 32 μg/ml | 30 (19.2) | 10 (15.6) | 20 (21.7) |  |
| ≥ 64 μg/ml | 2 (1.3) | 1 (1.6) | 1 (1.1) |  |
| Clofazimine†, N (%) |  |  |  | 0.910 |
| ≤ 0.25 μg/ml | 19 (38.8) | 9 (42.9) | 10 (35.7) |  |
| 0.5 μg/ml | 26 (53.1) | 10 (47.6) | 16 (57.1) |  |
| ≥ 1 μg/ml | 4 (8.1) | 2 (9.5) | 2 (7.1) |  |

^*^The *P*-value was calculated based on the comparison of maintenance doses of clofazimine.

†The measurement of MIC for clofazimine was available in clinical isolates from 49 patients.

Supplementary Table 2. Predictive factors for culture conversion within 6 months and microbiological cure (univariate analysis)

|  | Culture conversion within 6 months | *P*-value | Microbiological cure | *P*-value |
| --- | --- | --- | --- | --- |
| Age, years | 0.97 (0.94-1.00) | 0.024 | 0.96 (0.93-0.99) | 0.014 |
| Male sex | 0.50 (0.26-0.96) | 0.038 | 0.44 (0.22-0.88) | 0.021 |
| BMI, kg/m^2^ | 1.10 (1.00-1.22) | 0.054 | 1.05 (0.95-1.15) | 0.374 |
| History of tuberculosis | 1.26 (0.57-2.77) | 0.569 | 1.41 (0.60-3.35) | 0.434 |
| COPD | 0.19 (0.02-1.62) | 0.129 | 0.16 (0.02-1.37) | 0.094 |
| Smear positivity | 0.41 (0.19-0.89) | 0.024 | 0.29 (0.13-0.65) | 0.002 |
| Presence of cavity greater than 2 cm | 0.54 (0.29-0.99) | 0.047 | 0.64 (0.34-1.21) | 0.167 |
| Species |  |  |  |  |
| *M. avium* | Reference |  | Reference |  |
| *M. intracellulare* | 0.46 (0.23-0.89) | 0.022 | 0.75 (0.38-1.48) | 0.404 |
| Others | 0.97 (0.38-2.51) | 0.955 | 1.97 (0.67-5.78) | 0.217 |
| Clofazimine maintenance dose |  |  |  |  |
| 50 mg | Reference |  | Reference |  |
| 100 mg | 0.73 (0.39-1.35) | 0.315 | 1.03 (0.54-1.97) | 0.928 |

Abbreviations: BMI, body mass index; COPD, chronic obstructive pulmonary disease.
